# Elite Suppressors Harbor Low Levels of Integrated HIV DNA and High Levels of 2-LTR Circular HIV DNA Compared to HIV+ Patients On and Off HAART

**DOI:** 10.1371/journal.ppat.1001300

**Published:** 2011-02-24

**Authors:** Erin H. Graf, Angela M. Mexas, Jianqing J. Yu, Farida Shaheen, Megan K. Liszewski, Michele Di Mascio, Stephen A. Migueles, Mark Connors, Una O'Doherty

**Affiliations:** 1 Department of Pathology and Laboratory Medicine, University of Pennsylvania School of Medicine, Philadelphia, Pennsylvania, United States of America; 2 The Center for Aids Research, University of Pennsylvania School of Medicine, Philadelphia, Pennsylvania, United States of America; 3 Biostatistics Research Branch and NIAID, National Institutes of Health, Bethesda, Maryland, United States of America; 4 Laboratory of Immunoregulation, NIAID, National Institutes of Health, Bethesda, Maryland, United States of America; Fred Hutchinson Cancer Research Center, United States of America

## Abstract

Elite suppressors (ES) are a rare population of HIV-infected individuals that are capable of naturally controlling the infection without the use of highly active anti-retroviral therapy (HAART). Patients on HAART often achieve viral control to similar (undetectable) levels. Accurate and sensitive methods to measure viral burden are needed to elucidate important differences between these two patient populations in order to better understand their mechanisms of control. Viral burden quantification in ES patients has been limited to measurements of total DNA in PBMC, and estimates of Infectious Units per Million cells (IUPM). There appears to be no significant difference in the level of total HIV DNA between cells from ES patients and patients on HAART. However, recovering infectious virus from ES patient samples is much more difficult, suggesting their reservoir size should be much smaller than that in patients on HAART. Here we find that there is a significant difference in the level of integrated HIV DNA in ES patients compared to patients on HAART, providing an explanation for the previous results. When comparing the level of total to integrated HIV DNA in these samples we find ES patients have large excesses of unintegrated HIV DNA. To determine the composition of unintegrated HIV DNA in these samples, we measured circular 2-LTR HIV DNA forms and found ES patients frequently have high levels of 2-LTR circles in PBMC. We further show that these high levels of 2-LTR circles are not the result of inefficient integration in ES cells, since HIV integrates with similar efficiency in ES and normal donor cells. Our findings suggest that measuring integration provides a better surrogate of viral burden than total HIV DNA in ES patients. Moreover, they add significantly to our understanding of the mechanisms that allow viral control and reservoir maintenance in this unique patient population.

## Author Summary

Although HIV infection usually results in a syndrome of immunodeficiency, there is a small group of people infected with the virus who naturally control the infection. These subjects (called elite suppressors) are capable of mounting a more effective immune response against the virus, which maintains virus levels below detectable limits without clearing the infection. When plasma virus levels are this low, however, it becomes difficult to determine how much virus is present, and whether there are changes in viral burden over time. Here we show that measuring the level of integrated HIV DNA in this patient population provides valuable information regarding the mechanisms involved in viral control. Specifically, we show that levels of integrated HIV DNA are much lower in elite suppressors compared to other patients on and off anti-retroviral drugs, consistent with having a smaller reservoir. We show that 2-LTR circles, comprised of DNA that is replication incompetent, are much more common in elite suppressors. Thus, we identified two important differences between elite suppressors and other HIV infected individuals. We further show that ES cells appear to be just as susceptible to HIV integration as those from uninfected donors, suggesting that the elevated 2-LTR circles are not the result of an inherent restriction to integration. We conclude that measuring integration of HIV DNA in patient samples may provide a surrogate measure of viral burden in these and other patients with HIV.

## Introduction

A small percentage (less than 0.5%) of people who are infected with Human Immunodeficiency Virus (HIV) are capable of naturally controlling the infection without the use of highly active anti-retroviral therapy (HAART) [1]–[7]. These patients, termed elite suppressors (ES), are seropositive but maintain viral RNA levels in plasma below 50–75 copies per mL [1]. The mechanisms involved in viral control may include a combination of viral [8], [9] and host-mediated factors [10]–[12], which appear to be variable between patients [2], [4]. This group, therefore, is likely comprised of a heterogeneous population of people controlling virus replication by different mechanisms [3], [13] and to different extents [14]. Accurate and sensitive methods to measure very low viral burden by different parameters are needed to further characterize this patient population and may help identify subsets within this category.

Viral burden in HIV infected patients can be measured as viral particles containing RNA, cell associated viral RNA, and total and integrated viral DNA. Viral burden quantification in ES has been limited to measurements of viral RNA in plasma, cell-associated viral RNA or total DNA (often described as “proviral DNA”) in PBMC, and estimates of Infectious Units per Million cells (IUPM) [14]–[17]. To our knowledge, there have been no previous attempts to specifically measure integrated DNA in ES patients. Integrated viral DNA is believed to be of great importance in the establishment of a latent reservoir that is resistant to HAART and measuring integration may serve as a surrogate measure of the viral reservoir in the absence of ongoing replication [18]. The establishment of this latent reservoir is thought to occur early in the course of infection [19], but the contribution of integrated HIV DNA to viral persistence in ES patients remains unknown. Here, we apply a unique, sensitive and precise method to measure integrated HIV DNA in PBMC samples obtained from ES patients. In order to accurately measure very low levels of HIV integration in this cohort, we increased the sensitivity of our previously described repetitive sampling *Alu*-*gag* PCR integration assay [20] by increasing the number of genomes assayed per well. We find low but measurable levels of integrated DNA in 10 out of 10 ES patients with a wide distribution of levels of integration between patients. The level of integration in ES patients was significantly lower than that in equally suppressed patients on HAART. This is intriguing, given that we and others [17] find the level of total HIV DNA in ES patients is similar to that in HAART treated patients, albeit with small data sets for comparison. To determine if the unintegrated HIV DNA in the ES samples was due to the accumulation of 2-LTR circular DNA, we measured 2-LTR circular HIV DNA in these (and other) samples. We found 2-LTR circles were present at detectable levels more often in samples from ES patients, compared to samples from patients on HAART and off HAART. Furthermore, the levels of 2-LTR circular HIV DNA were higher in ES than in on-HAART patients. As shown through *in vitro* inoculations, the excess of 2-LTR circles was not due to an innate restriction at the level of integration in the ES cells since integration occurred with similar efficiency in CD4+ T cells from ES and normal donors after *in vitro* inoculation with HIV.

## Results

### Generation and validation of our quantitation methods for HIV Integration (Figure 1)

**Figure 1:**
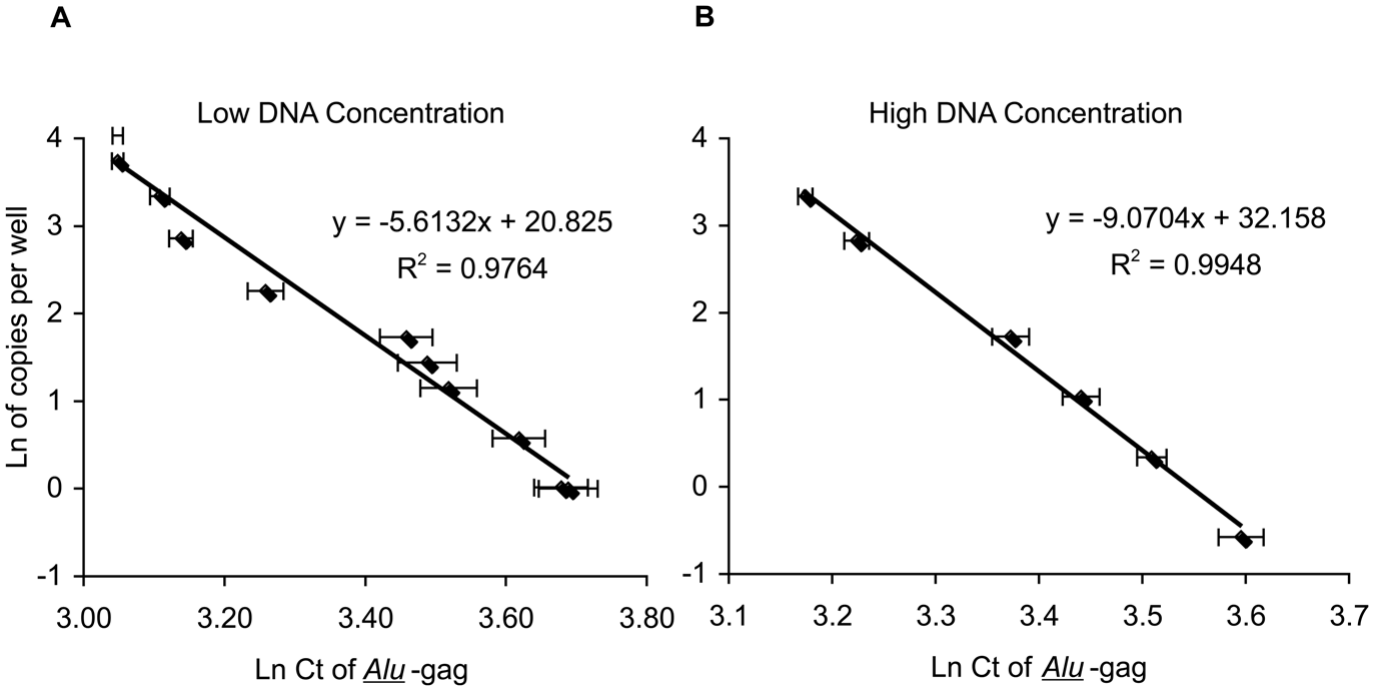
Linear correlation between Ln (Ct of *Alu*-*gag*) and Ln (HIV DNA copies per well) at low and high DNA concentrations. The integration standard was diluted in PBMC DNA from HIV-negative donors at 2 (**A**) or 40 (**B**) µg/ml as indicated for each standard curve in order to obtain samples containing known numbers of integrated HIV DNA copies. 25 µl of the standard were then assayed per well after adding 25 µl of the PCR master mixture for a total of 50 µl in each reaction. The final concentration of HIV DNA in each well is therefore, 1 and 20 µg/ml for the low and high DNA concentrations, respectively. Each point represents the average Ln(Ct) for 42 replicates.

As previously described, a linear correlation exists between the average natural log of the *Alu*-*gag* cycle thresholds (Cts), and the natural log of the number of integrated HIV DNA copies per well (Figure 1A). Using these methods we can detect down to 1 copy of HIV DNA integrated in 15,000 genomes (7,500 diploid cells). To enhance the sensitivity of our assay in order to measure lower integration levels, we simply added more genomes to each well and created a new standard curve for samples of DNA at 300,000 genomes per 50 µl reaction (Figure 1B). We needed to generate a new standard curve at the higher genome number because as expected, *Alu-gag* signal per integration level is weaker at higher genome copies. This is expected because increased genomes will result in a larger number of Alu-Alu competitive reactions. As before, we then calculate the average level of integration and the 95% confidence interval using the standard curve and the 42 replicates as described [20]. To use this method, we require that the average *Alu*-*gag* Ct is lower than the average *gag*-only Ct. At lower levels of integration, the average *Alu*-*gag* Cts were not statistically different than the *gag-*only Cts. However, we found that individual *Alu*-*gag* Cts were clearly significantly different than the *gag*-only signals. Thus, we found that we could correlate the percent positive *Alu*-*gag* signals to the proviral number as described in detail in Figure S1, allowing us to quantify even lower integration values.

We validated both methods of quantitation (*Alu*-*gag* Ct values vs percent positive signals) by demonstrating that we could achieve similar measurements for a given sample using both standard curves (Table S1). Additionally, we validated the reproducibility of both methods by measuring the same sample on different days (Table S1, samples D and E). Finally, we determined the coefficient of variation for total and integrated DNA measurements by preparing DNA from 6 separate aliquots of PBMC. Each total and integrated HIV DNA measurement was performed on different days by 3 different individuals (Table S2).

### HIV DNA Integration levels in ES patient samples (Figure 2)

Using the above methods designed to measure integration in patients with extremely low levels of integrated HIV DNA, we assayed ten elite suppressors (Table 1) at 300,000 genomes per 50 µl. Integration was measurable in all ten elite suppressor samples tested. The median level of integration (+/−S.D.) for the cohort was 14.4, +/−16.0 integrated HIV DNA copies per million PBMC. Integrated HIV DNA levels ranged from one copy in 19,000 cells all the way down to around one copy in 2 million cells. For 4 of the 10 patients, it was necessary to use the percent positive method at 300,000 genomes in 50 µl due to the low level of integrated HIV DNA in the sample (patients 1, 5, 6 and 8) as described in Figure S1. For the remaining 6 patients, the average Ct method was valid.

**Figure 2:**
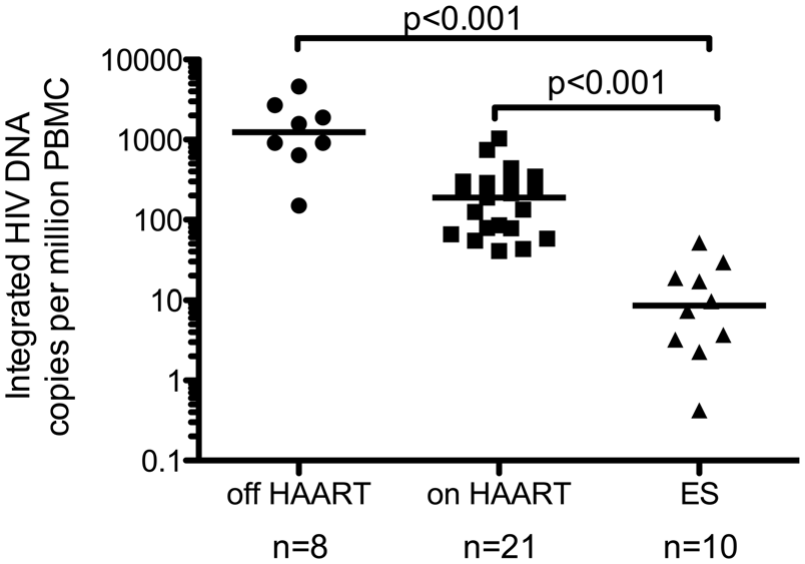
Elite suppressors have lower levels of integration than other HIV+ patients on and off HAART. HIV DNA Integration levels from ten ES patients were compared to the levels found in 21 patients on and 8 patients off HAART. Individual levels of total and integrated HIV DNA have been previously published for 10 of the patients on and 6 of the patients off HAART [21], [22]. The level of integration in ES patients was significantly lower than patients off and on HAART. The level in the on HAART group was also statistically lower than off HAART (p<0.001, statistical test described in methods). The line represents the median value.

**Table 1:** Elite suppressor characteristics.

Patient number	Viral load (copies/mL)	Year of diagnosis	CD4+T cell count (cells/µL)
1	8	1985	1232
2	1.5	1985	892
3	16.3	1998	1616
4	<1	1989	745
5	<1	1996	997
6	1	1991	643
7	<1	1987	655
8	<50	1985	679
9	<50	1997	559
10	<50	1987	884

### Elite suppressors have smaller reservoirs than HAART treated patients (Figure 2 and Figure 3)

The levels of integrated HIV DNA found in 10 elite suppressors (Table 1) were compared to the levels in 21 HAART treated subjects and 8 patients off HAART. Measurements from six of the off HAART patients and ten of the on HAART patients were previously published in Yu et al. [21] and measurements from seven of the on HAART patients were previously reported in Agosto et al. [22]. Table S3 reports the characteristics for the remaining patients (4 on and 2 off HAART). All HAART treated patients had viral loads below the limit of detection and the CD4^+^ T cell counts for the cohort were not statistically different from those of the elite suppressors (nonparametric Wilcoxon rank-sum test p>0.05). Although both patient populations have undetectable viral loads, the level of integrated HIV DNA in elite suppressors is significantly lower than that found in HAART treated patients (Figure 2, p< 0.001), consistent with a smaller and difficult to measure reservoir size (as had been previously assessed by attempts to culture out infectious virus [15], [17]). For a subset of ES and on HAART patients we were able to normalize our data to CD4^+^ T cell counts and to µL of blood. The level of integrated HIV DNA per CD4^+^ T cell or per µL was still significantly lower in the ES group compared to patients on HAART (Figure S2 A,C).

**Figure 3:**
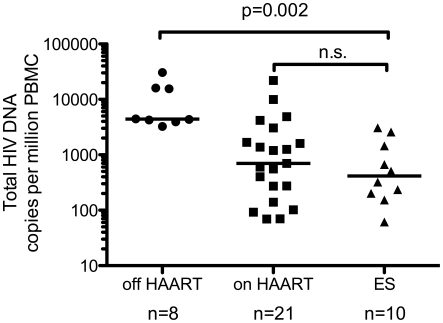
Total HIV DNA is not significantly different in patients on HAART and ES patients. Total HIV DNA was measured by RU5 quantitative PCR in PBMC from 2 non-controllers off and 4 on HAART and elite suppressor patients. Serial two-fold dilutions (from 1e6 to 2.5e5 cells per well) were measured in triplicates for each sample. This was repeated on a separate extraction for all ES. In all cases inhibitors were demonstrated to be absent by dose response in 3 serial two-fold dilutions. Data from 6 off and 17 on HAART patients previously measured was also included for analysis [21], [22]. Patients off HAART have higher levels of total HIV DNA than other patient populations. The level of total HIV DNA was not significantly different between patients on HAART and ES, while both were significantly lower than patients off HAART (p = 0.002 for both). The line represents the median value.

Samples were also subjected to PCR targeting the RU5 region as a measure of total HIV DNA. Intriguingly, HIV RNA/mL of plasma [14], [16] and total HIV DNA per cell [17] are similar in these two patient populations. We confirmed this comparison within this sample population, noting no significant difference in the level of total HIV DNA between ES patients and patients on HAART (Figure 3). In addition, when normalized to CD4^+^ T cell count or µL of blood, the level of total HIV DNA is still not statistically different between patients on HAART and ES, although the median level is lower in ES (Figure S2 B,D).

### Measures of total and integrated HIV DNA in ES patients show a large excess of unintegrated HIV DNA (Figure 4)

**Figure 4:**
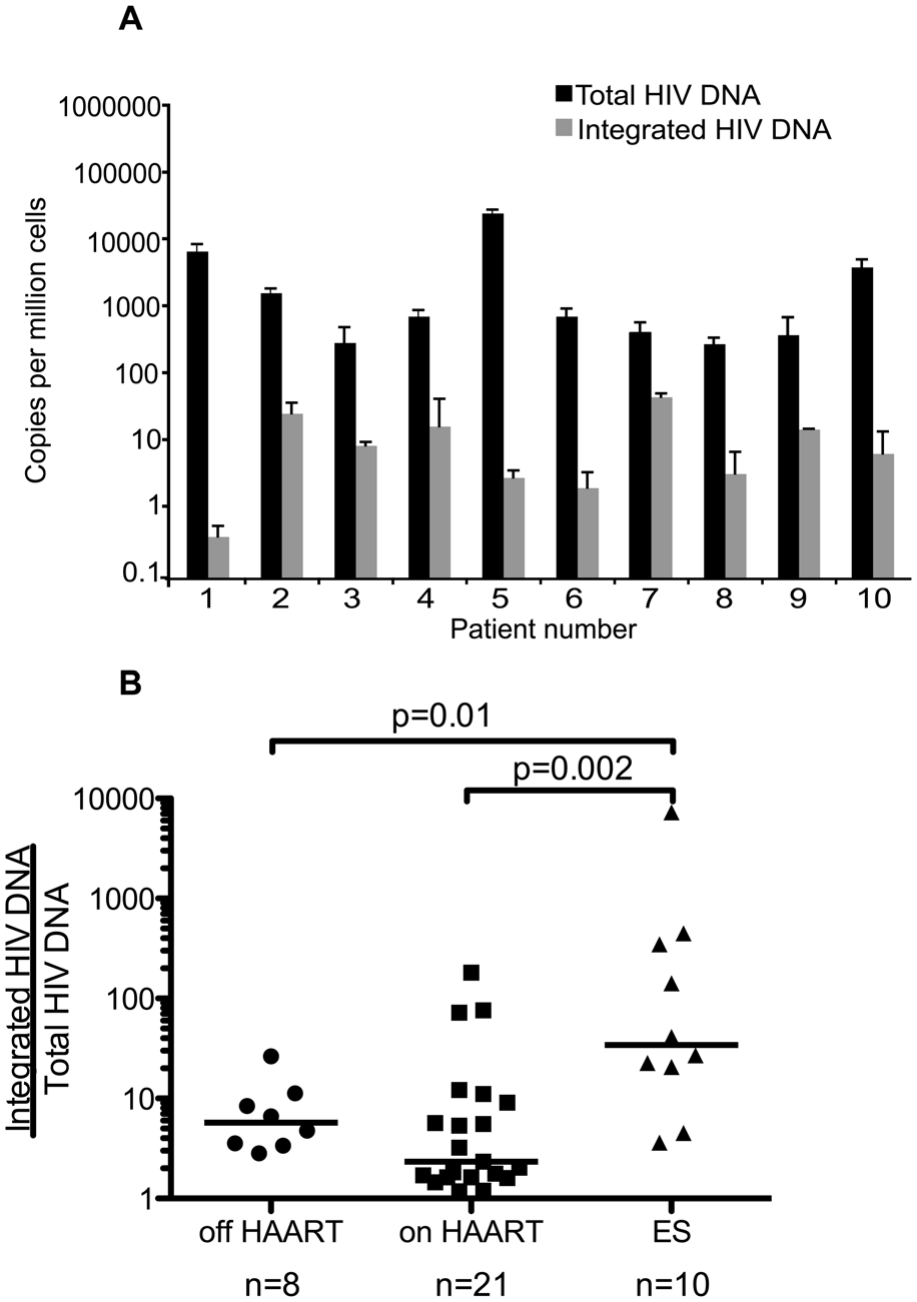
ES patients have a large excess of unintegrated HIV DNA. Total and integrated HIV DNA was compared in ES patients (**A**). The ratio was then calculated by dividing the number of total HIV DNA copies per cell by the number of integrated HIV DNA copies per cell for each patient sample, and these were compared to patients on and off HAART. The excess of unintegrated HIV DNA was significantly higher in the ES patient population than either patients on or off HAART (**B**). However, the levels were not different between patients on and off HAART (p = 0.172). The line represents the median value.

Previous measures of total HIV DNA in ES patients showed similar levels in ES patients and patients on HAART [17] and our data support this conclusion (Figure 3). A few studies suggest total may be slightly lower in ES [2], [23] thus a larger number of samples might reveal total HIV DNA is slightly lower in ES. Here we show in 10 ES patients there is a large difference between the levels of total and integrated HIV DNA in the ES patient population (Figure 4 A,B), suggesting that measuring total HIV DNA is not a suitable estimate of the size of the HIV reservoir in ES. We also compared the ratios of total vs. integrated HIV DNA measured in each patient sample, as an estimate of excess unintegrated HIV DNA, and found a significant difference between ES patients and patients on and off-HAART (Figure 4B).

### 2-LTR circles in patient samples (Figure 5)

**Figure 5:**
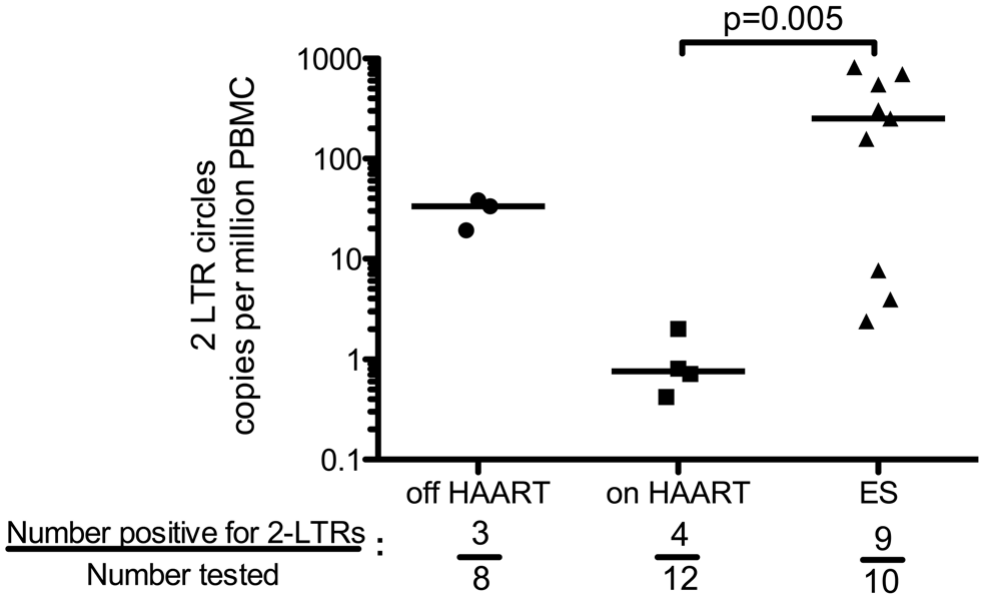
ES patients harbor higher levels of 2-LTR circles than HIV+ patients on HAART. DNA samples from ten ES patients were analyzed for 2-LTR circular HIV DNA. 2-LTR circular HIV DNA levels were compared to those in samples from HIV+ patients on and off-HAART. Only ES and on HAART could be compared due to small samples size. The level of 2-LTR circles was higher in ES as was the frequency with which they were detected (p = 0.007, chi-square test). The line represents the median value.

In order to determine if the excess of unintegrated HIV DNA found in ES patients is due to the accumulation of circular DNA intermediates we measured 2-LTRs in each patient sample. We assayed samples from 12 of the 21 patients on HAART, 8 patients off HAART, and 10 ES patients for 2-LTR circles using 1 million cells per replicate in at least three wells. We detected 2-LTRs in 4/12 (33%) of the patients on-HAART, 3/8 (37.5%) of the patients off-HAART and 9/10 (90%) of the ES patient samples. Figure 5 shows the median and standard deviation of the 2-LTR levels for the samples in which we detected signals in each patient population. The median level of circular 2-LTR DNA in ES patients is significantly higher than that in the on HAART patient population. Our ability to detect 2-LTR HIV DNA is consistent with previous reports in the literature [24]–[27] and suggests that 2-LTR circles are a minor component in chronic progressors on and off-HAART. However, when we assayed 2-LTR circles in the ES samples, we detected them in 9 of the 10 patients we sampled and the median levels were higher than those found in patients on HAART, by Wilcoxon rank-sum test (Figure 5).

### Excess of 2-LTR circles in ES are not explained by innate restriction to integration (Figure 6 and Table 2)

**Figure 6:**
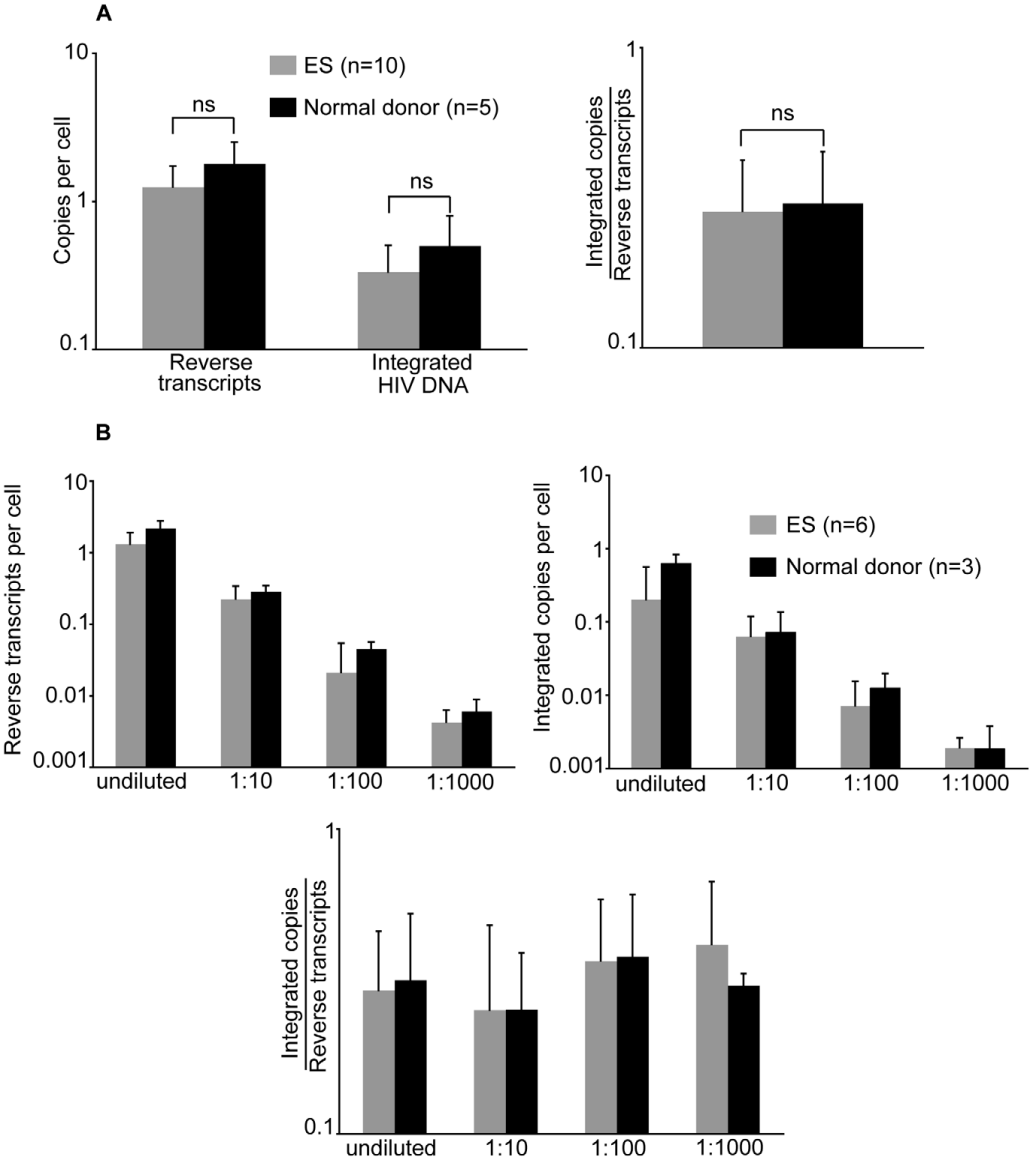
ES CD4^+^ T cells are not resistant to *in vitro* infection. CD4+ T cells from 10 ES and 6 uninfected donors were inoculated with NL4-3 (**A**). Reverse transcripts and integrated HIV DNA were measured at 2 days post-infection. From these values, integration efficiency was calculated (*i.e.* integrated copies per reverse transcript). ES were compared to normal donors with a Wilcoxon rank-sum test. Neither reverse transcription, integration nor integration efficiency measurements were statistically different between the two populations. Six ES were also inoculated with 3 serial ten-fold dilutions of the same virus (**B**). The bars represent the averages of multiple experiments (n is indicated in figures) and the error bars represent the standard deviations.

To examine whether the high levels of 2-LTR circles found in ES could be explained by a block to infection after reverse transcription but prior to integration, ES cells were infected *in vitro*. We negatively isolated unstimulated CD4^+^ T cells, by bead depletion, from all 10 ES samples, and spinoculated with an X4-tropic virus (NL4-3) in the presence of saquinavir. As controls, cells from uninfected donors were inoculated side by side with the ES cells. At two and four days post-inoculation, we isolated DNA and measured reverse transcription and integration. The levels of reverse transcription and integration were not statistically different when compared with normal donors two days (Figure 6A) and four days (data not shown) after infection. Moreover, the integration efficiency was the same between the two populations as shown by integrated copies per reverse transcript (Figure 6A). To ensure that ES did not possess a restrictive factor that was oversaturated at a high MOI, we also infected 6 of the 10 ES samples with serial dilutions of virus, down to a 1∶1,000 dilution. Consistent with the high MOI data (Figure 6A), the dilution series also yielded similar levels of reverse transcription, integration, and integration efficiency (integrated copies per reverse transcript) between the normal donor and ES cells (Figure 6B). In Table 2 we compared the percent of HIV DNA that was integrated between the ES *in vitro* experiments at a 1∶1000 dilution (to mimic *in vivo* levels of infection) and ES *in vivo* measurements. We find that ES cells contain a lower percentage of integrated HIV DNA *in vivo* when compared to the levels after *in vitro* inoculation (Table 2). The lower percentage found *in vivo* in ES is consistent with the idea that these cells are under stronger immune pressure. A CCR5 tropic HIV was also tested in the same system, yielding similar results (data not shown). These results argue against the presence of an innate restriction to integration in ES cells that would result in high levels of 2-LTR circles.

**Table 2:** Percentage of integrated HIV DNA per reverse transcript.

	ES CD4+ T cells	Normal Donor CD4+ T cells	
*in vitro*	42	31	
	ES Patients	Off HAART	On HAART
*in vivo* (HIV+)	6.6	19.2	39

## Discussion

A small percentage of people that become infected with HIV-1 are able to naturally control viral levels without the use of antiretroviral therapies. Understanding the mechanisms that enable control of viremia in these patients could further our understanding of the pathogenesis of this virus and aid in the development of vaccines against HIV-1. Since this population is defined by having undetectable virus levels in plasma (using conventional methods of detection), other methods need to be employed to measure differences in viral burden within this group. Our lab has developed a very accurate and sensitive method for quantitation of integrated HIV DNA in PBMC from patients with HIV [20], [21], [28] based on *Alu* repeats [29], [30]. Using a well-described integration standard we enhanced our methods of quantitation to measure very low levels of integration. We quantify low levels of integrated viral copies by increasing the number of genomes we assay per well. Furthermore, by counting the percent of positive *Alu*-*gag* signals after using our repetitive sampling *Alu*-*gag* PCR integration assay, we further lowered our limit of detection to enable quantitation of very low numbers of integrated HIV DNA copies in each patient sample. Using these methods we found that ES patients harbor lower levels of integrated HIV DNA compared to patients whose plasma viremia is equally suppressed by highly active antiretroviral therapy (HAART). This is an important difference between ES and patients on HAART given that viral RNA/ml [14], [16] and total DNA/cell [17] are similar in these two groups. We also found higher levels of 2-LTR circles in ES patient samples compared to samples from patients on HAART, which confirms our finding of a large excess of unintegrated HIV DNA in ES samples.

Elite suppressors and HAART treated patients are not distinguishable when some measures of viral burden are compared such as viral RNA in plasma and total HIV DNA copies in cells. Independent groups have compared viral RNA/ml of plasma in ES and patients on-HAART by these methods and found similar levels between these populations [12], [14]–[16], [31]. More recently, Julg *et al* compared total HIV DNA levels in ES and HAART treated patients and again found no significant differences in viral burden as assessed by total HIV DNA [17]; although though another study found slightly lower levels of total HIV DNA but did not do a direct comparison to patients on HAART [2].

One clear difference between ES and HAART treated patients is the size of the reservoir as estimated by using Infectious Unit Per Million (IUPM) assay. This method enumerates the number of latently infected cells, or the number of cells capable of producing infectious virus following stimulation of cells in vitro. Blankson *et al* used this approach to characterize replication competent HIV-1 in a subset of ES and was able to successfully culture 6 viral isolates from 4 ES. In this study, virus was not cultured from 6 of the 10 participants, making viral load quantification impossible in these patients. Nevertheless, the authors could conclude that the viral reservoir in ES must be at least one log lower than that of HAART treated patients using the IUPM assay. Julg *et al* also attempted to recover replication competent viruses from ES and were only able to detect replicating viruses in 2 of 14 ES sampled, even though they found equal numbers of total HIV DNA copies in ES and HAART treated patients [17]. In summary, viral quantitation in this patient population using the IUPM method was only possible in 6 out of 24 (25%) of the patients tested. Thus, viral reservoir size is smaller in ES compared to HAART patients, but it is difficult to measure viral reservoirs by the IUPM assay because the reservoir size is nearing detection limits for this assay.

Taken together, these prior studies point towards an important discrepancy between total HIV DNA/cell measures and reservoir size. Low levels of integrated HIV DNA in ES can explain the discrepancy between total HIV DNA and reservoir size. Total HIV DNA is made up of integrated and unintegrated HIV DNA forms. Unintegrated HIV DNA forms accumulate in cells when reverse transcription is completed but integration does not occur. 2-LTR circles are noted to accumulate to high levels when integration is inhibited [32]–[34]. Unintegrated HIV DNA (and especially circular forms which are a dead-end product) is thought to contribute minimally, if at all, to viral production [34]–[41]. Thus, it stands to reason that measuring integrated HIV DNA is a better surrogate of replication competent virus than total HIV DNA. Nonetheless, how good a surrogate integrated DNA is for reservoir size remains unclear.

There is data to suggest that integrated DNA may be a useful surrogate for reservoir size. For example, studies suggest that the reservoir size is constant over time [42], [43] and that the level of integrated HIV DNA is relatively constant over time ([44]–[47] and our unpublished data). The similar kinetic profile between these two parameters suggests they mimic each other. Here we find that integrated HIV DNA is very low in ES consistent with studies showing that reservoir size, as estimated by IUPM, is low in ES. Thus, our data further support that integrated HIV DNA may be a useful surrogate for reservoir size. Given the difficulty and cost to measure IUPM and that IUPM cannot even be detected in some ES, measurement of integration would be an attractive surrogate if valid. Notably, we successfully measured integration levels in all ES samples.

Measurements of integrated HIV DNA in ES may provide useful information and help characterize this unique group of patients. In one study, Hatano *et al* measured levels of HIV RNA in plasma, cell-based HIV RNA, and total DNA in patients with plasma levels <50–75 copies/ml who had not received antiretroviral therapies [16]. The authors showed a trend toward a slow increase in viral RNA levels, suggesting the reservoir size may increase over time. In addition, viral evolution is detectable in ES supporting the notion that ongoing replication is present in this patient cohort [48], [49]. Perhaps, by applying our methods of detection for integrated HIV DNA over time in ES we could detect small changes in viral reservoirs in this patient population. In another study, Sedaghat *et al* studied a cohort of ES over-time and failed to detect changes in CD4^+^ T cell counts over a 10-year period. However, they demonstrate in one patient that treatment with HAART led to a marked decrease in markers of immune activation [50]. Whether ES would benefit from therapeutic intervention is currently unknown, but more sensitive measures of changes in reservoir size over time may help us determine the effect of therapeutic interventions in this patient subset.

Our patient data raises the possibility that T cells in ES patients may restrict HIV before the step of integration. In other words, if integration occurs inefficiently then more 2LTRs will form. We found no obvious restriction to integration after in vitro inoculation of CD4+ ES cells. These results are consistent with studies that demonstrated the kinetics of spreading infection were similar in activated ES and normal donor cells [17], [51].

At least three possibilities exist to explain our *in vivo* observations. One, ES CD4+T cells contain a restrictive factor that limits infection. Two, the 10 ES patients possess defective viruses that are inefficient at infection. Three, cytotoxic T lymphocytes destroy infected cells thus lowering the level of integrated HIV DNA, while cells containing 2-LTR circles are preserved. We were only able to partially test the first of these theories in the current work. We find that after inoculation in vitro, even at very low multiplicities of infection, ES CD4+T cells have similar integration efficiencies compared to normal donors. Defects at other steps of life cycle may still exist within ES cells and have only been partially addressed by the literature. Consistent with our data, spreading infection occurs with similar efficiency in activated PBMC from ES and normal donors; however, these cells were artificially activated and so may not be representative of cells in vivo [51], [17]. Notably, it was recently reported at International AIDS Symposium by Lichterfeld *et al*. that some steps in the viral life cycle appear to be restricted in HIV inoculated cells from ES patients, suggesting cellular restriction factors should not be completely ruled out. At the same time a recent report shows ES cells expressed similar levels of GFP after infection with an R5 or X4 pseudotyped GFP vector [52]. Therefore, if restrictions exist they likely involve accessory proteins that were not present in the recent report [52]. In conclusion, our data suggests that there is not a restriction at the step of integration. However, there are limitations to our and others' *in vitro* experiments. For example, it remains possible that there are restrictions at other steps in the viral life cycle. It is also possible that primary isolates would show a restriction not apparent with NL4-3 or AD8.

The lack of a restriction to integration leads us to suspect that CTL pressure on infected cells in ES individuals may eliminate cells with successful integration leading to lower levels of integrated HIV DNA. Strong CTL activity could keep the level of integration low by targeting cells that contain integrated HIV DNA if integration leads to the expression of HIV specific proteins that can be processed and presented at their surface. Several studies show CTL responses are more effective in ES than other HIV-infected individuals [12], [51], [53]-[61]. In addition, cells that contain 2-LTR circles should be under less CTL pressure as they express less HIV proteins. Furthermore, they may accumulate to higher levels because ongoing replication persists in ES [48], [49]. To understand the accumulation of 2-LTR circles in ES it may be important to determine the half-life of T cell subsets in ES patients given the low level of activation compared to progressors [62] and possibly patients on HAART [63].

In conclusion, we developed and validated a method of quantitation that is accurate and sensitive enough to measure very low levels of integrated HIV DNA in patient samples. Using this method we can more accurately measure reservoir size in a wide range of patients, including ES patients, patients on HAART and others. We find that ES patients harbor a smaller reservoir of integrated HIV DNA than well-suppressed patients on HAART. These patients harbor high levels of 2-LTR circular HIV-DNA, confirming that there is an excess of unintegrated HIV DNA in this patient population. Our findings add significantly to our understanding of the mechanisms that allow viral control in this unique patient population.

## Materials and Methods

### Integration assay

The two-step repetitive sampling integration assay for patient samples has been previously described [20]. Briefly, a first step PCR reaction containing a forward primer specific for the human *Alu* element and a reverse primer specific for the HIV *gag* gene is followed by a nested, real-time PCR reaction containing primers and a probe specific for the HIV LTR, allowing for quantification by cycle threshold values. Samples are analyzed in a minimum of 42 replicates [21]. Samples are simultaneously analyzed by the same method using only *gag* primers to account for background signals from unintegrated HIV DNA. We require a minimum of 5 positive signals to quantify each sample. Therefore, the number of repeats assayed varied from patient to patient, but was always greater than or equal to 42 replicates. Each patient is assayed on different days and on occasions it is required that we assay one patient on multiple different days to obtain robust measurements of integration (i.e. 5 positive wells). Therefore to control for day to day variation, we run 4 IS control samples on each run and require that the Ct values for these samples fall within a small range.

### Measurement of total HIV DNA

PCR amplification targeting the RU5 region of HIV was performed using the same primers and probe used in the second step of the integration reaction [21] in a single round of 40 cycles. DNA samples were diluted to contain 1e6, 5e5, and 2.5e5 cells per well for analysis in triplicate wells of a 96 well plate. Ct values were inserted into a regression line equation obtained from a standard curve from the same reaction using samples with known copy numbers of pNL4-3 plasmids. The mean and standard deviation of the nine measurements were calculated for each sample.

### High DNA concentration standard curve (300,000 genomes per well)

In order to enhance our ability to detect integrated DNA in patient samples with extremely low levels, the number of genomes assayed per well was increased from 15,000 genomes (7,500 cell equivalents) to 300,000 genomes (150,000 cell equivalents) per reaction. Our previous measurements were performed by diluting DNA samples to a concentration of 2 µg/mL or 15,000 genomes in 25 µL (low concentration) and then adding 25 µL of master mix. We chose a new concentration of 40 µg/mL or 300,000 genomes per 25 µL to establish a new standard curve. The final volume for each reaction is 50 µL/well, resulting in a final concentration of 1 µg/mL (low concentration) or 20 µg/mL (high concentration) of sample DNA.

### Analysis of integration level using Ln(Ct) vs Ln(HIV copies per well)

Using dilutions of our integration standard, previously described [28], in PBMC DNA and the repetitive sampling method, a standard curve was generated by plotting the natural log of the average cycle threshold versus the natural log of the number of HIV DNA copies per well at 300,000 genomes per well in order to calculate a new regression equation. The dynamic range of the high genome assay using this method is from 3.33 to 185 integrated HIV DNA copies per million cells. The top dilution of the standard was chosen based on the detection limit of our prior assay. When levels of integration are above this range our standard integration assay provides robust measurements and is the preferred method as it requires much less DNA [21].

### Analysis of integration level using percent of positive signals vs HIV copies per well

Standard curves were also generated by plotting the percent positive versus the integration levels for both the low (15,000 genomes per well) and high (300,000 genomes per well) DNA concentrations of genomes. The upper limit for the low genome curve is 133 integrated HIV DNA copies per million cells (or about 1 provirus per well with 7,500 cells) and the upper limit for the high genome curve is 16.6 integrated HIV DNA copies per million cells (or about 2.5 proviruses per well with 15,000 cells). The upper limit for these standard curves is caused by the plateau that occurs when 100% of wells are positive. In general, we only use repetitive sampling when less than 10% of the wells give a positive signal. The lower limit is defined by the number of replicate assays that are performed and so theoretically there is no lower limit. This analysis was used when either the average Ct for the *Alu*-*gag* signal was not significantly different than the *gag*-only signal, or when there was a limited amount of sample to assay as the percent positive method is more sensitive.

### Study subjects and ethics statement

Subjects were recruited by Connors and Migueles from the Clinical Research Center, National Institutes of Health (Bethesda, MD) and signed informed consent forms were approved by the National Institute of Allergy and Infectious Diseases Investigational Review Board. The University of Pennsylvania's Institutional Review Board approved the transfer and use of materials for this research. Frozen PBMC from ten elite suppressors (Table 1) were available for analysis. The patients are part of a cohort studied at NIH under protocol # 02-I-0086 and provide pheresis products every 6 to 12 months for research use [5], [31]. Each patient maintained a viral load below the limit of detection (<50 copies per mL) for at least eleven years post-diagnosis (median 22 (range 11–31) years) and all were either HLA-B*57 or B*27 positive. Median CD4 counts were 884 (range 559-1616) cells/µl and median CD8 counts were 707 (range 300–1118) cells/µl. PBMC were thawed and genomic DNA was extracted using the Blood and Cell Culture Maxi Prep Kit (Qiagen, Valencia, CA). DNA samples were analyzed for total, integrated, and 2-LTR circular HIV DNA.

We have previously published measurements of total and integrated HIV DNA in samples from 17 patients on and 6 patients off HAART [21], [22]. We have since measured total and integrated HIV DNA in two more patients off and four more patients on HAART (from NIH, Table S3). We used these measurements for comparisons with our new ES patient samples. The patients referred to as being off HAART had never been treated with antiretroviral therapies (treatment naïve). The 2-LTR circular HIV DNA for this study had not been previously published.

### Assay for circular 2-LTR HIV DNA

HIV 2-LTR segments were amplified using a kinetic PCR assay and methods previously described [64]. Serial dilutions of the 2-LTR plasmid of 1∶3 were preformed in a background of uninfected PBMC DNA. The dilutions were assayed from 1000 copies/well down to 1.4 copies/well. Using this method a positive signal from wells containing 1.4 copies could be detected 77% of the time, consistent with what would be predicted by the Poisson distribution. Samples containing 1.4 copies had the largest coefficient of variation (1.29).

### *In vitro* infection of CD4+ enriched PBMC from ES and HIV- donors

PBMC from all ten ES were stained with FITC labeled lineage markers for CD8, CD14, CD16, CD20, CD56, CD11c. CD4^+^ T cells were negatively selected following the anti-FITC bead depletion protocol (Miltenyi Biotec, Germany). CD4^+^ T cells were spinoculated with pNL4-3 virus at a high MOI (such that there was on average 1–2 reverse transcripts per cell). In addition, samples from 6 of the 10 ES individuals were spinoculated with 3 serial ten-fold dilutions of virus. Cells from HIV-negative donors, at equal cell numbers, were infected in parallel as controls. After spinoculation, cells were treated with saquinavir to inhibit spreading infection. At two and four days post-infection, DNA was isolated with the DNA micro kit (Qiagen, Valenica, Ca). Reverse transcription was measured using the same primers used for patient samples, targeting the RU5 region, and integration was measured by our *in vitro* integration assay (as described in [65]). The nonparametric Wilcoxon rank-sum test was used to compare measurements of reverse transcription and integration in the ES samples to those from the HIV-negative donor samples. A p-value of <0.05 would have been considered significant.

### Statistical analyses

The nonparametric Wilcoxon rank-sum test was used for unpaired comparisons. The associations between variables were assessed using the Spearman's rank correlation test or simple linear regression analysis. Chi-square test was used to assess whether differences in the percentage of LTR positive samples among groups were statistically significant. P values <0.05 were considered to be statistically significant. Statistical analyses were performed using WinSTAT.

## Supporting Information

Figure S1 Generation of standard curves using percent positive quantitation. At very low levels of integrated HIV DNA, the average signal from the integration-positive wells overlaps with the average signal from the control wells. In other words, the difference between the Ct values is no longer statistically significant (t-test p≥0.05). Consequently, we used a new method of quantitation that distinguishes the positive signals from background and uses the percent of positive signals to determine the number of integrated HIV DNA copies in a sample of cells. Dilutions of our polyclonal integration standard were used to define the correlation between the percent of wells with positive signals and the number of copies of integrated HIV DNA per well. The standard, which has one copy of integrated HIV DNA per cell distributed randomly to mimic a patient infection, was tested at 8 different dilutions from five copies per well down to 0.1 copies per well using the repetitive sampling method. Each dilution was made with 2 and 40 µg/mL PBMC DNA (final concentrations 1 and 20 µg/mL), isolated from HIV-negative donors, to yield a range of integration levels. The percent of positive wells was then plotted against the number of copies of integrated HIV DNA per well to calculate a regression equation. Error for this assay is calculated by the quotient formula (p(1-p)) for the Poisson distribution. **A** The integration standard was diluted in PBMC DNA and assayed for *Alu*-*gag* and *gag*-only using repetitive sampling PCR as described at high, intermediate and low number of integration events per well. The top paired panels are the typical PCR curves generated when there are high levels of integrated HIV DNA in a patient sample, the average Ct value of *Alu*-*gag* signals is always lower than that of *gag*-only signals and there is less variability between Ct values for individual wells. The middle panel shows the PCR curves generate at intermediate levels of integrated HIV DNA. Here, there is greater variability between individual Ct values due to the array of distances between *Alu* and gag in any given sample and some of the signals overlap with *gag*-only controls. However the average *Alu-gag* is still significantly lower than the gag-only. Finally, the bottom panel shows the PCR curves when there are low levels of integrated HIV DNA. In this case, the average *Alu-gag* and average gag only are not statistically different. Nonetheless, positive signals are still detected outside of the *gag*-only range. In this final situation, we must use the percent positive method to detect integration as described below. **B,C** There is a linear correlation between the percent of positive signals and the number of integrated HIV DNA copies per well at both 1 (low DNA concentration, **B**) and 20 (high DNA concentration, **C**) µg/mL of DNA. The standard curve was generated by diluting IS in a constant number of uninfected cells. PCR was performed on multiple replicates at each concentration. The percentage of positive wells at any dilution was determined and plotted against the known number of integration events. To determine the number of positive wells, the average Ct +/- two standard deviations from the *gag*-only signal are applied to the *Alu*-*gag* signals to determine if the difference between *Alu*-*gag* and *gag*-only signals is significant (p<0.05).
(1.33 MB TIF)

Figure S2 Integrated and Total HIV DNA measurements normalized to CD4+T cell count and uL of blood. For 11 of the patients on HAART and all 10 of the ES patients tested, we were able to normalize the measurements of integrated HIV DNA and total HIV DNA to copies per million CD4+T cells (**A**,**B**) and copies per uL of blood (**C**,**D**). The level of integrated HIV DNA was still significantly lower in ES compared to patients on HAART (**A**,**C**) and total HIV DNA was still not statistically different between the two groups (**B**, **D**) by either method of normalization. The lines represent the median values.
(0.15 MB TIF)

Table S1 Validation of quantitation methods: Both assays provide similar integration levels when tested in patient samples. Five patient samples were measured at both (low and high) DNA concentrations, and the average levels of integrated HIV DNA measured are shown. At these concentrations, the samples required quantitation by the percent positive method at the low DNA concentration and the average Ct method at the high DNA concentration. D and E were measured in triplicate, while A, B and C were single measurements due to limiting sample. When compared at the two concentrations, the measurements for D and E were similar.
(0.03 MB DOC)

Table S2 Reproducibility of total and integrated HIV DNA assays. The same patient sample was assayed for total and integrated HIV DNA separately, a total of 6 times. Each assay was performed by a different person twice, so that 3 people independently measured the sample for each intermediate.
(0.03 MB DOC)

Table S3 Characteristics of additional patients on and off HAART.
(0.03 MB DOC)
