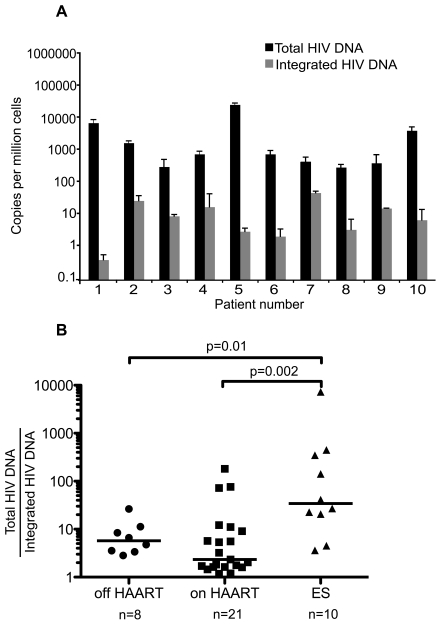# Correction: Elite Suppressors Harbor Low Levels of Integrated HIV DNA and High Levels of 2-LTR Circular HIV DNA Compared to HIV+ Patients On and Off HAART

**DOI:** 10.1371/annotation/0d21de23-d44c-49c0-9a9f-53d421648cbf

**Published:** 2011-03-24

**Authors:** Erin H. Graf, Angela M. Mexas, Jianqing J. Yu, Farida Shaheen, Megan K. Liszewski, Michele Di Mascio, Stephen A. Migueles, Mark Connors, Una O'Doherty

Figure 4 is incorrect. The y-axis of Figure 4B was mislabeled. It should be "Total HIV DNA/Integrated HIV DNA," but it was labeled the inverse. Please view the correct Figure 4 here: 

**Figure:**