# Supplementary figures and images for: Elite Suppressors Harbor Low Levels of Integrated HIV DNA and High Levels of 2-LTR Circular HIV DNA Compared to HIV+ Patients On and Off HAART
Source: PLoS Pathog. 2011 Feb 24;7(2):e1001300. doi: 10.1371/journal.ppat.1001300 (PMC3044690; doi:10.1371/journal.ppat.1001300)

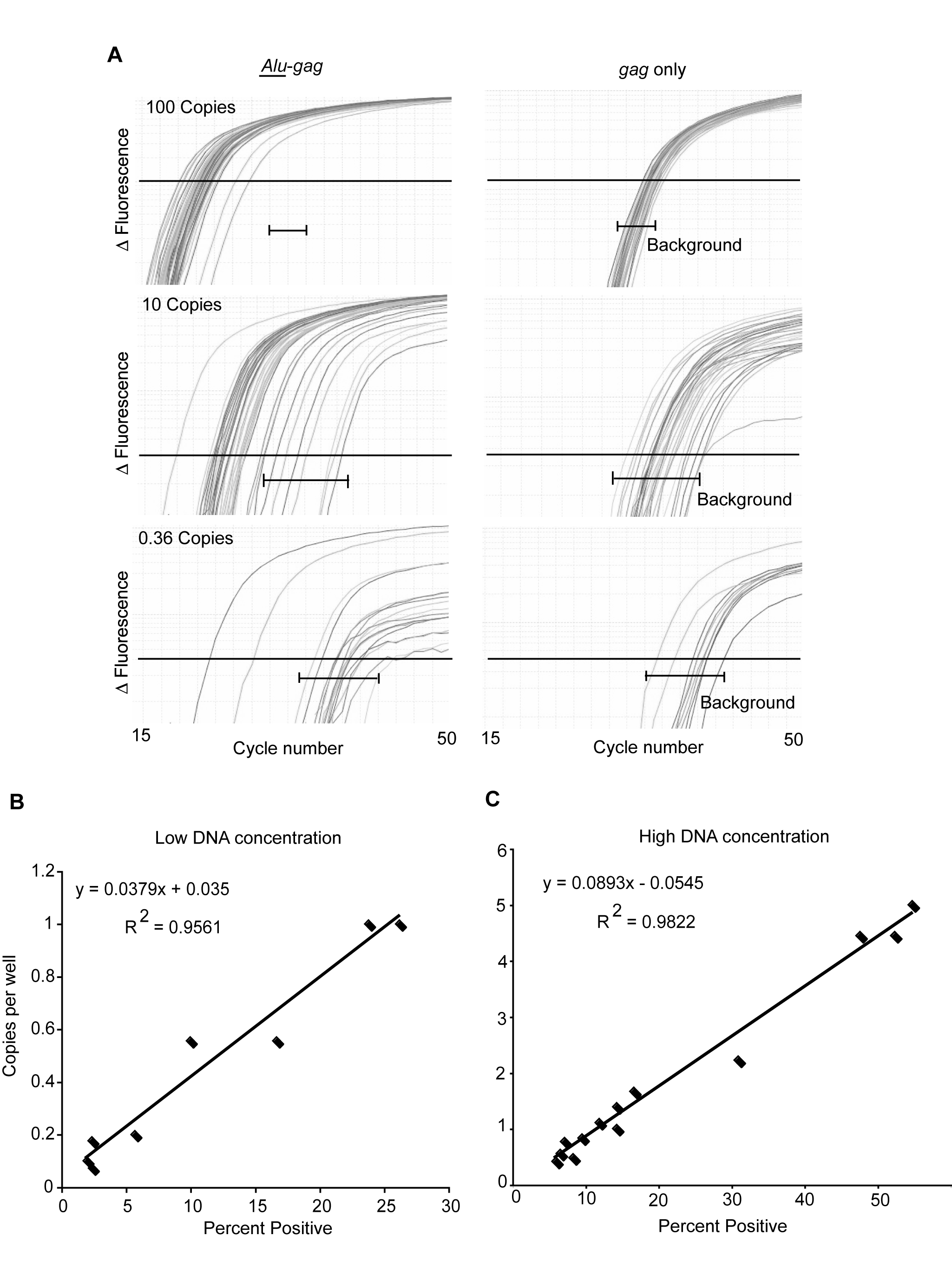

Supplement: Figure S1 — Generation of standard curves using percent positive quantitation. At very low levels of integrated HIV DNA, the average signal from the integration-positive wells overlaps with the average signal from the control wells. In other words, the difference between the Ct values is no longer statistically significant (t-test p≥0.05). Consequently, we used a new method of quantitation that distinguishes the positive signals from background and uses the percent of positive signals to determine the number of integrated HIV DNA copies in a sample of cells. Dilutions of our polyclonal integration standard were used to define the correlation between the percent of wells with positive signals and the number of copies of integrated HIV DNA per well. The standard, which has one copy of integrated HIV DNA per cell distributed randomly to mimic a patient infection, was tested at 8 different dilutions from five copies per well down to 0.1 copies per well using the repetitive sampling method. Each dilution was made with 2 and 40 µg/mL PBMC DNA (final concentrations 1 and 20 µg/mL), isolated from HIV-negative donors, to yield a range of integration levels. The percent of positive wells was then plotted against the number of copies of integrated HIV DNA per well to calculate a regression equation. Error for this assay is calculated by the quotient formula (p(1-p)) for the Poisson distribution. A The integration standard was diluted in PBMC DNA and assayed for Alu-gag and gag-only using repetitive sampling PCR as described at high, intermediate and low number of integration events per well. The top paired panels are the typical PCR curves generated when there are high levels of integrated HIV DNA in a patient sample, the average Ct value of Alu-gag signals is always lower than that of gag-only signals and there is less variability between Ct values for individual wells. The middle panel shows the PCR curves generate at intermediate levels of integrated HIV DNA. Here, there is greater var [file ppat.1001300.s001.tif]

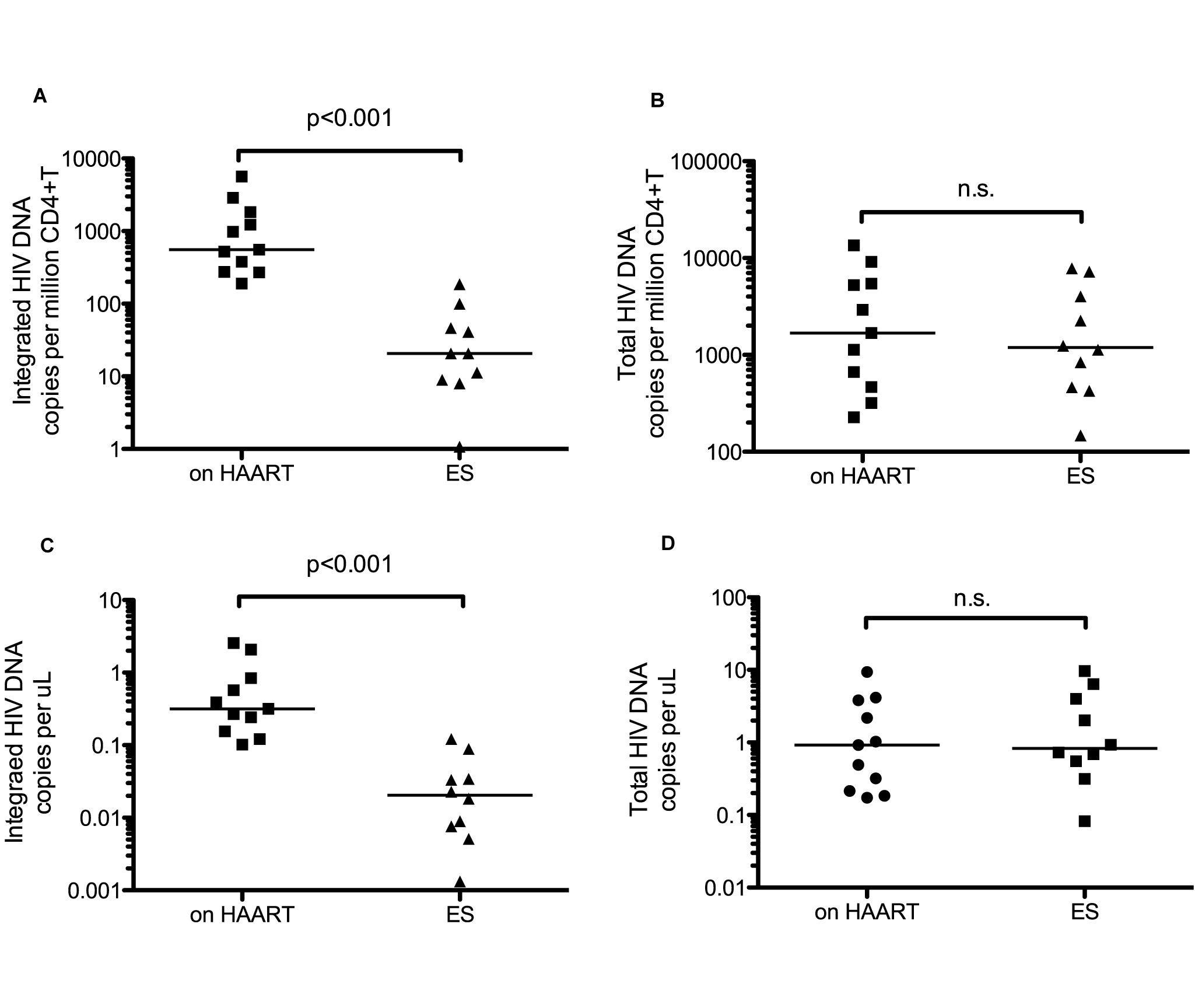

Supplement: Figure S2 — Integrated and Total HIV DNA measurements normalized to CD4+T cell count and uL of blood. For 11 of the patients on HAART and all 10 of the ES patients tested, we were able to normalize the measurements of integrated HIV DNA and total HIV DNA to copies per million CD4+T cells (A,B) and copies per uL of blood (C,D). The level of integrated HIV DNA was still significantly lower in ES compared to patients on HAART (A,C) and total HIV DNA was still not statistically different between the two groups (B, D) by either method of normalization. The lines represent the median values. (0.15 MB TIF) [file ppat.1001300.s002.tif]
