# Supplementary material for: Elite Suppressors Harbor Low Levels of Integrated HIV DNA and High Levels of 2-LTR Circular HIV DNA Compared to HIV+ Patients On and Off HAART
Source: PLoS Pathog. 2011 Feb 24;7(2):e1001300. doi: 10.1371/journal.ppat.1001300 (PMC3044690; doi:10.1371/journal.ppat.1001300)
Supplement: Table S1 — Validation of quantitation methods: Both assays provide similar integration levels when tested in patient samples. Five patient samples were measured at both (low and high) DNA concentrations, and the average levels of integrated HIV DNA measured are shown. At these concentrations, the samples required quantitation by the percent positive method at the low DNA concentration and the average Ct method at the high DNA concentration. D and E were measured in triplicate, while A, B and C were single measurements due to limiting sample. When compared at the two concentrations, the measurements for D and E were similar. (0.03 MB DOC) [file ppat.1001300.s003.doc]

Table S1. Validation of quantification methods: both assays provide similar integration estimates when tested in patient samples.

| Patient | Integrated HIV DNA measured at high concentration* | Integrated HIV DNA measured at low concentration* | p-value |
| --- | --- | --- | --- |
| A^#^ | 31 | 27 | - |
| B^#^ | 10 | 9.4 | - |
| C^#^ | 53 | 51 | - |
| D | 40 +/-5.7 | 41 +/-42 | 0.97 |
| E | 13 +/-2.3 | 17 +/-14 | 0.85 |

* Copies per million cells +/- standard deviation

^#^ Quantities were limiting, therefore samples could not be measured multiple times as with D and E
