# Supplementary material for: Elite Suppressors Harbor Low Levels of Integrated HIV DNA and High Levels of 2-LTR Circular HIV DNA Compared to HIV+ Patients On and Off HAART
Source: PLoS Pathog. 2011 Feb 24;7(2):e1001300. doi: 10.1371/journal.ppat.1001300 (PMC3044690; doi:10.1371/journal.ppat.1001300)
Supplement: Table S2 — Reproducibility of total and integrated HIV DNA assays. The same patient sample was assayed for total and integrated HIV DNA separately, a total of 6 times. Each assay was performed by a different person twice, so that 3 people independently measured the sample for each intermediate. (0.03 MB DOC) [file ppat.1001300.s004.doc]

Table S2. Reproducibility of total and integrated HIV DNA measurements

| Repeat | Total HIV DNA  (copies per million cells) | Integrated HIV DNA  (copies per million cells) |
| --- | --- | --- |
| 1 | 520 | 180 |
| 2 | 450 | 160 |
| 3 | 440 | 140 |
| 4 | 630 | 110 |
| 5 | 440 | 120 |
| 6 | 440 | 120 |
| Average +/-stdev | 487+/-77 | 138+/-27 |
