# Supplementary material for: Elite Suppressors Harbor Low Levels of Integrated HIV DNA and High Levels of 2-LTR Circular HIV DNA Compared to HIV+ Patients On and Off HAART
Source: PLoS Pathog. 2011 Feb 24;7(2):e1001300. doi: 10.1371/journal.ppat.1001300 (PMC3044690; doi:10.1371/journal.ppat.1001300)
Supplement: Table S3 — Characteristics of additional patients on and off HAART. (0.03 MB DOC) [file ppat.1001300.s005.doc]

Table S3. Characteristics of additional patients on and off HAART

| Patient Number | HIV-RNA  (copies/mL) | CD4+ T cell count (cells/mL) | Years with <50 on ART |
| --- | --- | --- | --- |
| 11 | <50 | 578 | 3 |
| 12 | <1 | 1409 | 13 |
| 13 | 2 | 372 | 7 |
| 14 | <50 | 453 | 4 |
| 15 | 78,211 | 609 | untreated |
| 16 | 273,450 | 516 | untreated |
